# Circulation and Seasonality of Respiratory Viruses in Hospitalized Patients during Five Consecutive Years (2019–2023) in Perugia, Italy

**DOI:** 10.3390/v16091394

**Published:** 2024-08-30

**Authors:** Alessandro Graziani, Silvia Bozza, Monica Borghi, Antonella Mencacci, Barbara Camilloni

**Affiliations:** 1 Microbiology and Clinical Microbiology Section, Department of Medicine and Surgery, University of Perugia, 06132 Perugia, Italy; alessandro.graziani@dottorandi.unipg.it (A.G.); silvia.bozza@unipg.it (S.B.); antonella.mencacci@unipg.it (A.M.); 2 Microbiology Unit, Santa Maria della Misericordia Hospital, 06132 Perugia, Italy; 3 Istituto Zooprofilattico Sperimentale dell’Umbria e delle Marche, 06126 Perugia, Italy; m.borghi@izsum.it

**Keywords:** respiratory viruses, circulation, seasonality, surveillance

## Abstract

The emergence of SARS-CoV-2 and the non-pharmacological interventions adopted to counter its spread appear to have led to changes in the normal circulation and seasonality of respiratory viruses. Our study aims to investigate changes related to the circulation of respiratory viruses, not SARS-CoV-2, among hospitalized patients in Perugia, Central Italy, between 2019 and 2023. The samples were collected from individuals who went to the emergency room (ER) or were hospitalized and analyzed using a molecular multiplex test. The results underline that non-pharmaceutical interventions altered the typical seasonal circulation patterns of different respiratory viruses. Those mostly affected were enveloped viruses like influenza viruses that disappeared in 2021; the least impact was recorded for Rhinovirus, which was detected during the pandemic period, maintaining the same seasonality observed in the pre-pandemic period although with a reduction in the number of positive samples. Our data underline the importance of the continuous monitoring of these viruses, especially to understand the timing with which prevention measures, not only non-pharmacological interventions but also the equipment of vaccine doses and monoclonal antibodies, should be adopted to reduce their circulation, particularly in the population at risk of developing severe forms of lower respiratory tract infection.

## 1. Introduction

Respiratory viruses have always represented an important public health problem due to their contagiousness [1]. Lower respiratory tract infections (LRTIs) are considered an important cause of morbidity and mortality, especially in young adults and the elderly, where viral infections are responsible for community-acquired pneumonia in 20–40% and 50% of cases, respectively [2].

Historically, the most common viruses that can cause Severe Acute Respiratory Infection (SARI) are the influenza virus (FLU) in adults and the Respiratory Syncytial Virus (RSV) in children, but there are many other viruses responsible for the burden of this disease, such as Rhinovirus (RHV), Adenovirus (AdV), Parainfluenza virus (PIV), human Metapneumovirus (hMPV), Enterovirus (ENV), Bocavirus (BoV), and human Coronaviruses (hCoVs) [3,4]. Recently, the Acute Respiratory Syndrome Severe Coronavirus 2 (SARS-CoV-2) has been added to these viruses. It appeared in China in December 2019 [5] and spread globally to the point of being responsible for a pandemic.

Respiratory viruses have a specific seasonality pattern that depends on geographic zones and climate conditions [6]. In temperate regions, FLU, RSV, and hCoV show an incidence peak during the winter season, while AdV, hMPV, BoV, and RHV can be detected throughout the year, reaching a peak in the spring or autumn period [7]. Generally, in temperate climates, outbreaks of viral respiratory infection occur during winter, whereas in summer, the activity of these viruses is reduced [8].

The rise of SARS-CoV-2 and the non-pharmacological interventions (NPIs) adopted to contrast its spread, such as travel restrictions and mask wearing, appear to have led to changes in the normal circulation and seasonality of respiratory viruses [9,10]. During the pandemic period, a significant reduction in influenza virus activity was reported worldwide due to NPIs and viral competition with SARS-CoV-2. However, the mitigation measures did not affect all respiratory viruses in the same way; in fact, a different epidemiological trend seems to have been found for non-enveloped viruses like RHV, which, after the easing of restrictions, started to be detected again [11].

Unlike other respiratory viruses, the virological surveillance of influenza viruses was already well organized before the pandemic. The control and surveillance of influenza has been carried out since 1952 through the Global Influenza Surveillance and Response System (GISRS) of the World Health Organization (WHO), in which the National Influenza Centers (NICs) play a crucial role [12]. With the aim of greater control of the spread of SARS-CoV-2 and its variants, many European countries have integrated this surveillance system with the analysis of the circulation of SARS-CoV-2 and other respiratory viruses. In Italy, epidemiological surveillance is carried out based on the reporting of flu-like syndromes by general practitioners (GPs), while virological surveillance is carried out on samples received from the regional reference laboratory, collected by hospital doctors and GP sentinels operating in the regional area [13]. From the pandemic onwards, the Italian surveillance system that previously monitored the circulation of influenza viruses extended its activities to all respiratory viruses and was called “RespiVirNet” [14].

The evaluation and full understanding of the circulation and seasonality of respiratory viruses can represent an important weapon in the prevention of these infections because it would allow health facilities to equip themselves, before the start of their circulation, with the necessary therapeutic and preventive measures, such as in the case of recently introduced vaccines and monoclonal antibodies for RSV [15].

This study aims to describe the circulation of respiratory viruses, not SARS-CoV-2, in patients hospitalized at Santa Maria della Misericordia Hospital (Perugia, Italy) between 2019 and 2023 to evaluate the effects of the COVID-19 pandemic on their circulation and seasonality.

## 2. Materials and Methods

We analyzed respiratory samples collected between 1 January 2019 and 31 December 2023 from people who accessed the emergency room or from hospitalized patients at Santa Maria della Misericordia Hospital (Perugia, Italy). The subjects showed at least one general and one respiratory symptom among the following: fever, fatigue, headache, myalgia or arthralgia, cough, sore throat, and breathlessness. The samples analyzed in our study may vary according to the patient’s characteristics (age, severity of symptoms, invasive mechanical ventilation) and used nasal swab (NS), pharyngeal swab (PS), sputum, bronchoalveolar lavage (BAL), tracheal aspirate (TA) and bronchoaspiration (BA).

Samples from the ER have been analyzed using a rapid RT-PCR molecular test Xpert^®^ Xpress CoV-2/Flu/RSV plus (Cepheid, Sunnyvale, CA, USA) that simultaneously detects SARS-CoV-2, influenza virus A and B, and RSV, providing a result in less than 45 min thanks to the integration of sample preparation, nucleic acid extraction, and amplification in a single cartridge ready to use. A Sample Processing Control (SPC) and a Probe Check Control (PCC) are included in the cartridge for the adequate processing of the sample and to monitor the presence of potential inhibitors in the RT-PCR reaction.

Samples from hospitalized patients have been analyzed by extracting nucleic acids with a STARlet automated instrument (Seegene, Seoul, South Korea ) and then by a one-step Real-Time PCR using the kit Allplex™ Respiratory Panel Assays 1–3 (Seegene, Seoul, South Korea), following the manufacturer’s instructions. The kit analyzes samples using a multiplex that can detect influenza A/H1N1pdm09, influenza A/H3N2, influenza B, RSV-A, RSV-B, Adenovirus, Enterovirus, Parainfluenza virus 1-4, Metapneumovirus, Bocavirus, Rhinovirus, and human Coronaviruses (229E, NL63, OC43).

## 3. Results

### 3.1. Sample Collection and Molecular Test Results

During the study period, we analyzed 11,337 samples. Table 1 summarizes the number of samples collected per year.

**Figure 1:**
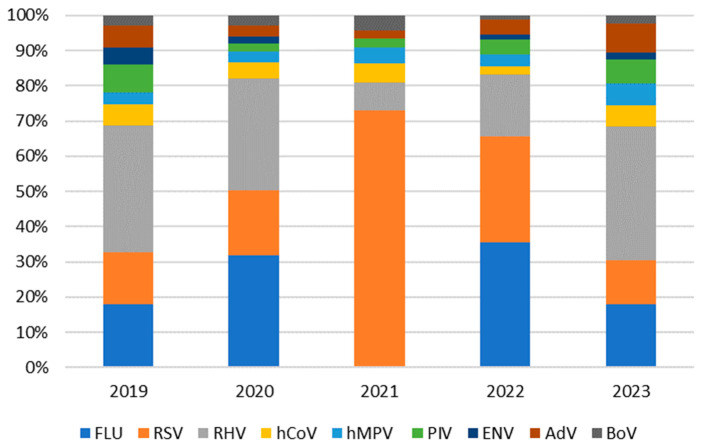
Positivity rates of each respiratory virus per year.

Of the 11,337 samples examined, 3936 (34.7%) were positive. In the 5 years of this study, as shown in Table 2 and Figure 1, the circulation of all respiratory viruses occurred, although at different levels, except for FLU and ENV, which disappeared in 2021.

### 3.2. Age Distribution

To examine the possible changes in respiratory viruses circulation among different age groups, we split the study subjects into five groups: 0–4 years, 5–14 years, 15–44 years, 45–64 years, and ≥65 years.

**Figure 2:**
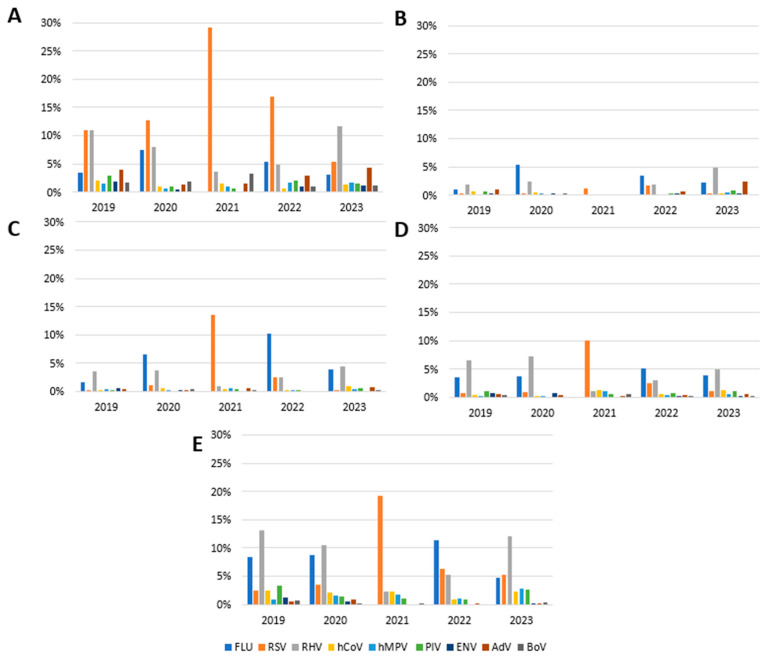
Annual positivity rate of each virus in different age groups: (**A**) 0–4 years; (**B**) 5–14 years; (**C**) 15–44 years; (**D**) 45–64 years; (**E**) ≥65 years.

For the 0–4 age group, it emerged that the percentage of samples positive for FLU was 3.4% in 2019, 7.5% in 2020, 5.4% in 2022, and 3.1% in 2023; in 2021, FLU was not detected. In this age group, the positivity rate for RSV was 11.0%, 12.6%, 16.9%, and 5.4% in 2019, 2020, 2022, and 2023 respectively, while we observed a peak of 29,1% in 2021. HRV showed a positivity rate of 10.9% in 2019, 8.0% in 2020, 3.7% in 2021, 4.9% in 2021, and 11.6% in 2023. Positive rates for other viruses like hCoV, MPV, PIV, ENV, AdV, and BoV decreased in 2020–2022 and returned to pre-pandemic levels in 2023 (Figure 2A).

In the 5–14 age group, we recorded an increased circulation of FLU in 2020 (5.4%) and 2022 (3.5%), while in 2021, FLU was not detected. The RSV incidence was very low in 2019 and 2020 (0.2%), increased in 2021 and 2022 (1.1% and 1.7%, respectively), and returned to 0.3% in 2023. HRV showed a rate between 0.2% in 2021 and 4.8% in 2023. No patient samples from this age group tested positive for hCoV in 2021 and 2022 and for PIV in 2020. hMPV was not detected in this age group in 2019, 2021, and 2022. ENV and BoV were not detected in 2021, as well as AdV, which was not detected in 2020 and which showed a positivity rate of 2.4% in 2023 (Figure 2B).

Regarding the 15–44 age group, the FLU circulation was variable and ranged from 0% in 2021 to 10.2% in 2022. RSV had a peak in incidence in 2021 with a positivity rate of 13.6%. hCoV, hMPV, and AdV were always detected in the years of the study with an incidence lower than 1%. PIV did not circulate in 2020, as well as ENV in 2021 and 2022 (Figure 2C).

The positivity rate of FLU in the 45–64 age group ranged from 3.6% to 5.1%, with no case occurring in 2021. The incidence of RSV was 0.7% and 0.9% in 2019 and 2020, with a peak of 9.9% in 2021. In 2022 and 2023, we saw a gradual return to pre-pandemic levels (2.5% and 1.1%, respectively). Rhinovirus had a positivity rate of 6.5% in 2019, 7.3% in 2020, 1.1% in 2021, 3% in 2022, and 4.9% in 2023. hCoV and hMPV had peak positivity during 2021 (1.3% and 1.1%, respectively), a percentage that also remained unchanged in 2023 for hCoV, compared to 0.4% in 2019. We have neither detected PIV and BoV in 2020 nor ENV in 2021. The positivity rate for AdV was 0.5% in 2019 and 0.6% in 2023, with a reduction in 2021 (0.2%) (Figure 2D).

For the age group ≥65, the positivity rate for FLU ranged from 8.5% in 2019 to 4.7% in 2023, with no case occurring in 2021. Regarding RSV, the positivity rate reached a peak of 19.2% in the year 2021 compared to 2.5% in 2019 and then remained at 5.2% in 2023. Rhinovirus had an incidence of 13.1% in 2019 and 10.5% in 2020, with a reduction in 2021 (2.2%) and then a recovery in the years 2022 and 2023 (5.3% and 12%, respectively). The hCoV detection was 2.5% in 2019, 2.1% in 2020, 2.2% in 2021, 0.9% in 2022, and 2.3% in 2023. The positivity rate of hMPV increased in this age group during the study period (from 0.9% in 2019 to 2.9% in 2023). We did not find ENV in 2021 and 2022; in 2023, we recorded a rather low incidence of 0.2% (compared to 1.3% in 2019). There were no positive cases reported for AdV in 2021 and for BoV in 2022 (Figure 2E).

### 3.3. Seasonality of Respiratory Viruses

**Figure 3:**
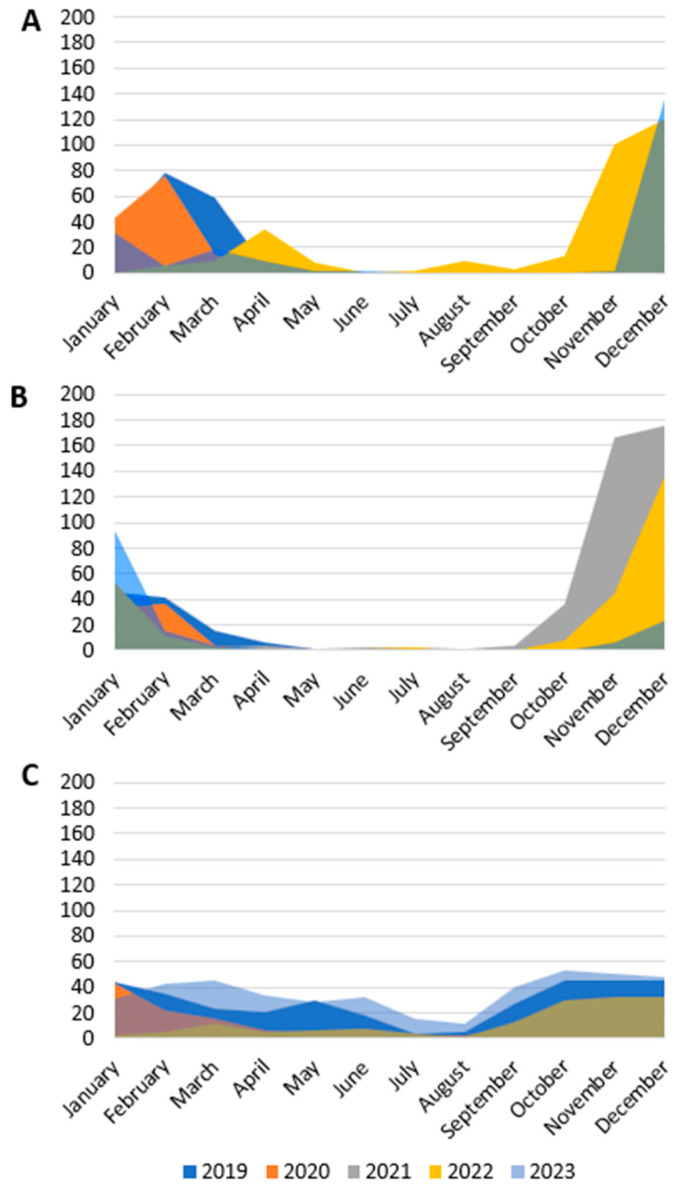
Monthly distribution of positive samples during the study period. (**A**) FLU; (**B**) RSV; (**C**) RHV.

To highlight any changes in the circulation of respiratory viruses, we have reported in Figure 3 the infections recorded monthly over the five years of this study.

In 2019, we observed the circulation of FLU between January and March. The same temporal trend was also observed in 2020; then, in 2021, FLU did not circulate. In 2022, FLU circulation was recorded from March to May and then in the summer with a peak of 9 cases in August; FLU was then detected in October (14 samples), November (100 samples), and December 2022 (120 samples). In 2023, positive samples for FLU were identified in January with 31 cases, in February with 8 cases, and in March with 18 cases, and then, it recurred again between November and December (138 cases) (Figure 3A).

In 2019, we detected RSV from January to March, and then in November and December; in 2020, we identified positive RSV samples only from January to March. In 2021, sporadic cases of infection were observed during the spring and summer months; the number of cases then increased from October to December. In 2022, RSV circulated in January and February; subsequently, circulation started again in October and the number of positive samples increased in the months of November and December. The same trend in circulation was observed in 2023 (Figure 3B).

RHV detection in 2019 occurred throughout the year with a peak between January and May and a reduced circulation in July and August. In 2020, the cases diagnosed were 43 in January, 35 in February, 16 in March, and then below 10 cases per month were detected in the following months (apart from 20 cases in October).

A small number of positive samples was observed in October 2021. In 2022, RHV infection was diagnosed every month, with a greater number of cases from October to December. In 2023, the diagnosis of RHV infection was reported every month with two peaks: in March and in October (Figure 3C).

**Figure 4:**
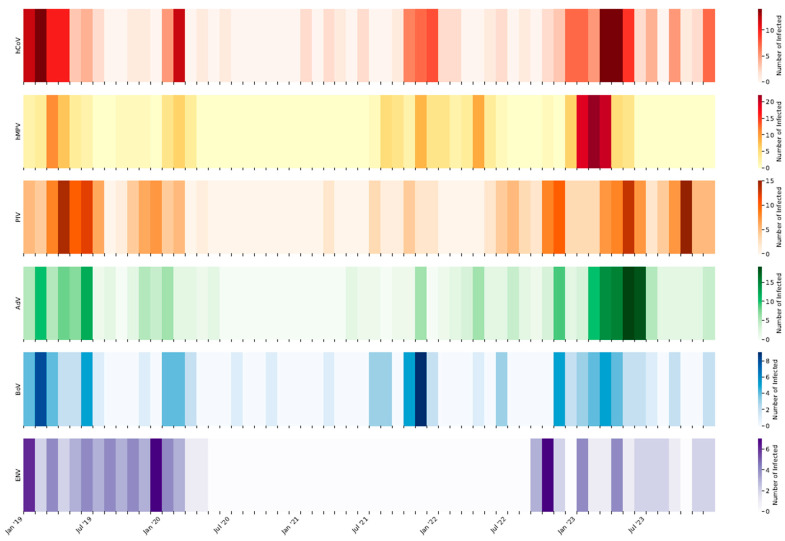
Number of positive cases detected each month of the study period for human Coronavirus (hCoV), human Metapneumovirus (hMPV), Parainfluenza virus (PIV), Adenovirus (AdV), Bocavirus (BoV), and Enterovirus (ENV).

Figure 4 shows the number of cases of the other respiratory viruses included in the study. The circulation of AdV, hCoV, and hMPV, usually concentrated in the first months of the year, suffered a strong decrease in 2020 and 2021. Their circulation resumed in 2022 and 2023 with the usual seasonal trend.

Before the pandemic, PIV circulated mostly in spring and fall. After the pause that characterized 2020 and 2021, their circulation resumed late in summer and autumn 2022 and in spring and autumn 2023, as in the pre-pandemic phase.

The circulation of BoV and ENV has always been rather limited, and before the pandemic, limited to the winter months. While ENV completely stopped its circulation from May 2020 to September 2022, BoV already resumed its circulation in autumn 2021. Between the end of winter and spring 2023, there was a rise in the circulation of BoV and ENV.

## 4. Discussion

In this descriptive study, we analyzed the impact of the COVID-19 pandemic and NPIs on the circulation and seasonality of non-SARS-CoV-2 respiratory viruses in Perugia hospital’s patients between 2019 and 2023. We chose 2019 as the reference year for the pre-pandemic period. In previous years, except for natural fluctuations, we did not find differences in the circulation of respiratory viruses when compared to 2019.

We analyzed 11,337 respiratory samples using different multiplex RT-PCRs depending on the department. We decided to perform a monotest assay (Xpert^®^ Xpress CoV-2/Flu/RSV *plus*) to analyze samples from the ER and kit that allowed batch testing (Allplex™ Respiratory Panel Assays 1–3) for all other departments. The first, compared to the other SOC tests, has 100% agreement for SARS-CoV-2, Flu A, and RSV and 99.4% for Flu B [16]; the second one is shown to be highly sensitive and specific and also a hands-on time-saving assay due to the automated nucleic acid extraction and PCR setup [17]. Moreover, the high-throughput batch testing may be useful to perform large numbers of assays and minimize the possibility of contamination since it is a one-step RT-PCR process that does not require the manipulation of post-amplification material [18].

Our study’s results highlight the absence of FLU in 2021 and a resumption of its circulation in 2022, although with an anomalous seasonality compared to previous seasons. The reduction in influenza virus activity in 2021 was documented in Italy by another research group [19], by the national surveillance system, and also in France and Spain, which did not mention positive samples during the 2020–2021 season [20]. The COVID-19 pandemic restrictive measures such as social distancing, facemask wearing, and school closures had an important effect, not only on SARS-CoV-2 containment but also on the circulation of other respiratory viruses. For example, influenza viruses disappeared in 2021 [21]. In 2022, we observed an unusual circulation of influenza viruses characterized by two peaks—in April and December—and with an out-of-season circulation during the summer, as also described in other Italian regions [6,22]. Unusual 2022 influenza virus circulation was also described in the Southern Hemisphere, in particular, in Australia [21].

Moreover, our data show a change in the circulation and seasonality of RSV during the pandemic. Particularly, we observed a greater positivity rate in 2021 compared to the pre-pandemic period, according to other studies conducted in Italy and in several other countries [23,24,25]. Moreover, in 2021, the circulation of this virus started between October and November, so earlier than the pre-pandemic period [23,26].

Historically, RSV infection has always been a public health problem, especially for children under two years of age [27]. This also emerged from our data, in which the 0–4-year age group was always the most affected during all the years of this study. In 2021, there was an increase in RSV circulation among age groups that were not usually characterized by widespread circulation. Similar results have also been shown in other Italian studies where a greater number of adults tested positive in that year [28,29].

The only exception concerns the 5–14-year age group, in which the incidence of RSV has always been the lowest, probably because these subjects have already had such infections not too long before or because they tend to be treated in outpatient settings.

The increased circulation of RSV was observed throughout Europe during 2021, the year in which RSV infected adults more than in previous seasons [28,30]. Many factors have probably contributed to this change in the circulation and seasonality of RSV: the NPIs adopted for the pandemic led to an important reduction in circulation during 2020; this change in RSV circulation is also probably connected to a competition between RSV and SARS-CoV-2. At the same time, these measures have determined a lack of population immunity to this virus that could explain the increased number of positive cases detected in 2021 [23,26]. Furthermore, we should also take into consideration that, after the pandemic, the testing strategy implemented for adult patients with respiratory symptoms has changed, and this may have contributed to a greater number of cases recorded [31].

Through the comparison of the two viruses, we can observe that FLU and RSV circulation was strongly reduced in 2020, and then, after the easing of the COVID-19 pandemic restrictions, a different timing with which the viruses returned to pre-pandemic circulation levels was observed: influenza virus resumed slowly to circulate again, while RSV had a rapid increase that led to an early and important diffusion in 2021 [11,22].

The circulation of hCoV during the pandemic suffered a significant reduction due to the adoption of containment measures. In the literature, the circulation of these viruses is described in the winter months followed by a reduction during the summer period [32]: also in our study, in fact, the circulation peak occurred both before and after the pandemic in the months between February and April.

In accordance with other studies [33,34], herein we found that hCoV most affected children under 4 years of age and subjects over 65. The trend that was observed before the pandemic was confirmed again in 2022 and 2023, following the easing of restrictive measures.

In our hospital, hMPV circulation occurred mainly in the spring, in both pre and post-pandemic phases, confirming what has been classically described in the literature [8].

In addition, our survey showed that after a reduction occurring between 2020 and 2021, hMPV returned to circulation at pre-pandemic levels in 2022, with a peak in cases reached in 2023. It is important to underline that the circulation of this virus usually begins at the end of RSV circulation, suggesting a possible interference between the two viruses [35,36].

Restriction measures also impacted the circulation of PIV, which was extremely reduced during the years of the pandemic. The age groups most affected by the infection are children under 4 years of age and adults over 65, and it is precisely in these age groups that the greatest effect of the adoption of NPIs is observed [37,38]. The seasonality of this virus remained unchanged before and after the pandemic, with the maximum circulation recorded in the spring and fall seasons, caused by a different strain of Parainfluenza viruses (PIV-3 in the spring and PIV-4 in the fall).

For non-enveloped respiratory viruses like RHV, a different impact of the mitigation measures on their circulation was observed [11]. In fact, we detected RHV during the pandemic period maintaining the same seasonality observed in the pre-pandemic period, although with a reduction in the number of positive samples. Other studies have shown that there is no viral interference between SARS-CoV-2 and RHV [36,39] and that non-enveloped viruses are more resistant to hydroalcoholic disinfectants, and their transmission is not prevented by masks [40,41,42]. On the contrary, we did not find Enterovirus in 2021. A comparison with other studies should be carried out, taking into account that some authors use kits that do not differentiate between RHV and ENV. For this reason, in some studies, the circulation of ENV may be overestimated [22,43,44]. Similar to our study, Klee et al. showed a strong decline in ENV circulation during the pandemic [38]. Furthermore, our data revealed a reduced circulation of this virus during the pandemic in subjects over 65 years of age, in accordance with Sclavi et al., who, through the sequencing of RHV/ENV samples, found that only a small number of elderly patients were positive for Coxsackievirus B [22].

Our results showed that AdV proved to be an agent responsible for respiratory tract infection, especially in children <14 years old, and BoV was mostly detected in children under 4 years of age, as already described in the literature [45,46]. Mitigation measures also impacted AdV and BoV circulation, which suffered an important reduction [38,47].

Overall, not all non-enveloped respiratory viruses have suffered the effects of the restrictive measures in the same way. If, on the one hand, these have not had a considerable impact on the circulation and seasonality of RHV, for others, especially EnV, the impact of NPIs was important [38]. Thus, a comprehensive explanation of our findings is still lacking, and further research is needed to better understand the circulation of enveloped and non-enveloped viruses during NPI.

This study has several limitations. First, as our research was a single-center study, there are regional and time limitations. Second, we analyzed a limited number of cases; as only patients with severe illness who required hospitalization were included in the study, this may not fully represent the community-level circulation of respiratory viruses. Thirdly, the distance between symptom onset and sampling was not standardized, and this could have impacted the detection rates of respiratory viruses. Furthermore, patients in the ER were only tested with the rapid molecular test Xpert^®^ Xpress, which is able to detect a limited number of viruses. This diagnostic approach may have led to an underreporting of co-infections if a further swab for respiratory viruses was not carried out during hospital admission. It is possible that the number of respiratory virus infections, other than SARS-CoV-2, has been underestimated during its maximum circulation. Once COVID-19 has been diagnosed, the patients may not have undergone further investigation to assess for other viruses.

## 5. Conclusions

This study underlines that NPIs, such as social distancing and wearing facemasks, play an important role in containing not only SARS-CoV-2 but also other respiratory viruses, affecting their typical seasonal circulation patterns. In particular, those most affected were enveloped viruses like influenza viruses that disappeared during the COVID-19 pandemic.

The relaxing of mitigation measures determined a resurgence of respiratory pathogens, also in out-of-season periods. So, it is very important to conduct surveillance programs for respiratory viruses and use multiplex panels to detect these pathogens even when it would not be expected to find them. Starting from the 2022–2023 season, for example, the Italian Network “InFluNet” was converted into “RespiVirNet”, extending the monitoring that previously covered only influenza to all other respiratory viruses. This new platform is an essential starting point to better understand the circulation of respiratory viruses, even for age groups that tend to be treated in outpatient settings, such as adolescents and young adults.

The continuous monitoring of these viruses is essential for implementing preventive measures and improving timing, as highlighted by the data from this study. In particular, they can optimize the use of NPIs; the purchase, stockpiling, and use of vaccines; and monoclonal antibodies that should be adopted to reduce their circulation, especially in the populations at risk of developing severe forms of LRTI.

The recent availability of vaccines and monoclonal antibodies for RSV prevention makes the collection of epidemiological data crucial and should influence decisions on the appropriate timing of prevention strategies.

## Figures and Tables

**Table 1:** Number of samples collected per year.

	Year				
Specimen Type	2019	2020	2021	2022	2023
**NS**	969	704	538	1206	2227
**PS**	1028	688	352	685	1709
**Sputum**	62	35	37	41	67
**BAL**	136	145	120	178	219
**TA**	16	47	14	12	33
**BA**	18	14	15	8	14
**Total**	2229	1633	1076	2130	4269

**Table 2:** Number of positive samples for each virus detected every year.

	2019	2020	2021	2022	2023
**FLU**	172	136	0	308	207
**RSV**	141	79	396	259	143
**RHV**	344	136	44	152	434
**hCoV**	57	19	29	20	70
**hMPV**	31	13	24	29	70
**PIV**	77	10	15	37	79
**ENV**	45	9	0	12	22
**AdV**	61	13	12	37	96
**BoV**	27	12	23	11	25
**Total**	955	427	543	865	1146

## Data Availability

The data are available upon request from the corresponding author. The data are not publicly available because of privacy or ethical restrictions.
